# A Phenomenological Paradigm for Empirical Research in Psychiatry and Psychology: Open Questions

**DOI:** 10.3389/fpsyg.2020.01399

**Published:** 2020-06-25

**Authors:** Leonor Irarrázaval

**Affiliations:** ^1^ Section Phenomenological Psychopathology and Psychotherapy, Psychiatric Department, University Clinic Heidelberg, Heidelberg, Germany; ^2^ Centro de Atención Psicológica, Facultad de Ciencias Sociales y Humanidades Sede Talca, Universidad Autónoma de Chile, Talca, Chile

**Keywords:** applied phenomenology, methodology, qualitative research, psychiatry and psychology, phenomenological interviews

## Abstract

This article seeks to clarify the way in which phenomenology is conceptualized and applied in empirical research in psychiatry and psychology, emphasizing the suitability of qualitative research. It will address the “What,” “Why,” and “How” of phenomenological interviews, providing not only preliminary answers but also a critical analysis and pointing to future directions for research. The questions it asks are: First, what makes an interview phenomenological? What are phenomenological interviews used for in empirical research in psychiatry and psychology? Second, why do we carry out phenomenological interviews with patients? Is merely contrasting phenomenological hypotheses or concepts enough to do justice to the patients’ involvement? Third, how should we conduct phenomenological interviews with patients? How can we properly perform analysis in empirical phenomenological research in psychiatry and psychology? In its conclusion, the article attempts to go a step beyond these methodological questions, highlighting the “bigger picture”: namely, the phenomenological scientific paradigm and its core philosophical claim of reality as mind-dependent.

## Introduction

An initial proposal in favor of “naturalizing phenomenology” was presented in the article “First-person methodologies: What, Why, How?” published by Varela and Shear (1999) in the *Journal of Consciousness Studies*. The authors were not only concerned with the need for a method in cognitive sciences to obtain empirically-based descriptions of the subject, but also with providing the basis for a “science of consciousness.” “Neurophenomenology” was proposed by Varela (1996) as a means of linking first‐ and third-person perspectives through a systematic examination of subjective experience within experimental settings. An important requirement of neurophenomenology was that both experimenter and experimental subject must learn the Husserlian phenomenological method. The notion “phenomenology” was employed in the etymological sense of the term, that is, “the study of that which appears” (from Greek *phainómenon* “that which appears” and *lógos* “study”). Additionally, Varela (1990) coined the term “enactive,” meaning not to act out or to perform as on a stage, but to “enact,” that is, “to bring forth” or to “emerge” (*hervorbringen*, in German), as it is used in the phenomenological tradition. Accordingly, the phenomenological method was conceived and applied as a form of training one’s attention to that which “appears” in the subject’s conscious experience, making it similar to a meditation technique. Examples of neurophenomenology are the experiments led by Lutz et al. (2002), which analyzed subjective reports, reaction times, and brain activity. However, a different approach was proposed by Gallagher (2003), who claimed that a “phenomenologically enlightened experimental science” means incorporating concepts and distinctions from the phenomenological analysis into the actual design of an experiment. In contrast to neurophenomenology, this approach does not require learning the Husserlian phenomenological method or even making first-person reports in the experiments. Examples of “front-loaded phenomenology” are neuroimaging experiments employing the phenomenological distinction between “sense of agency” and “sense of ownership” in involuntary movement (Ruby and Decety, 2001; Chaminade and Decety, 2002; Farrer and Frith, 2002).

However, experimental designs are normally not classified as part of qualitative research methodologies (Fischer, 2006; Maxwell, 2011, 2012; Patton, 2015; Creswell and Poth, 2018). One of the clearest differences between qualitative and quantitative approaches is that qualitative research is carried out in everyday natural conditions, rather than in experimental settings. Concerning the qualitative/quantitative distinction, there is an ongoing debate not only around the differences between the two approaches (Morgan, 2018; Maxwell, 2019), but also around whether they are actually distinguishable at all (Hammersley, 2018). Whatever their differences or similarities, qualitative and quantitative approaches are commonly conceived as compatible and their integration – in the form of mixed-methods research designs – valuable (Tashakkori and Teddlie, 2010). So, the incorporation of phenomenological interviews in experimental designs is one kind of mixed-method research design: One example is neurophenomenology, where the qualitative component is provided by phenomenology. Broadly speaking, qualitative research is used in many social sciences and humanities disciplines, including psychology, sociology, political sciences, and anthropology. A range of techniques are employed in qualitative research to gather experiential data, such as open-ended interviews, direct observation, focus groups, and document analysis (e.g., clinical records and personal diaries), and different methods are used for the associated qualitative data analysis, including phenomenology, ethnography, narrative analysis (e.g., biographical and life story studies), case studies, and grounded theory. In contrast to the large sample sizes needed in quantitative research to accomplish statistical validation of the results, qualitative research is characterized by an in-depth approach, which means working with few cases, with representativeness not being of such key importance (Barbour and Barbour, 2003). The use of less structured methods allows for the emergence of ideographic descriptions, personal beliefs and meanings, thus addressing the experiential processes of the subjects being studied (Schwartz and Jacobs, 1979; Barbour, 2000; Maxwell, 2011, 2012, 2019).

This article shall not focus on experimental phenomenology. However, this is no way meant to discredit in any sense this form of research design. Indeed, mention has already been made of the precursors of the experimental application of phenomenology to acknowledge the important contribution this research tradition has made – and continues to make – in ensuring that phenomenology acquires a scientific status. For instance, the project “cardiophenomenology” has been recently proposed by Depraz and Desmidt (2019) as a refinement of Varela’s neurophenomenology and performed in experimental studies of surprise in depression (Depraz et al., 2017). In addition, it is worth mentioning Martiny’s (2017) transdisciplinary research on the phenomenological and neurological aspects of living with brain damage, specifically cerebral palsy. Martiny’s work not only has been influenced by, but also seeks to revitalize, Varela’s “radical” proposal, reminding us of the importance of working with openness and a change of mindset in cognitive science. Usually framed as “embodied cognition,” this proposal approaches the mind as embodied, embedded, enacted, and extended (4E cognition), implying an awareness regarding the fact that the “embodied” notion applies not only to the mind of the experimental subject but also to the cognitive scientist carrying out the research (Depraz et al., 2003). Indeed, phenomenology has breached the frontiers of the philosophical discipline to influence the development of interdisciplinary fields of studies bridging the biomedical sciences and the humanities. Besides its application in the cognitive sciences, phenomenology is currently being widely applied in empirical research in healthcare-related disciplines, mostly in psychiatry and psychology. The most influential empirical application of phenomenology has been in the field of psychopathology, with the development of phenomenological interviews for the investigation of schizophrenia spectrum disorders (Parnas et al., 2005; Sass et al., 2017). However, the extent of phenomenology’s applicability outside the strict domain of philosophy is currently a topic of intense debate and controversy (Zahavi and Martiny, 2019). The conceptualization of phenomenology in the literature of qualitative research, which has been mostly developed in North America, is not always in line with that of the continental European philosophical tradition. Recent years have seen the start of a dialogue bridging the two traditions, qualitative research and philosophical phenomenology, giving a promise of fruitful collaboration in the future.

This article will address the “What,” “Why,” and “How” of phenomenological interviews, reviewing recent empirical research in the field of phenomenological psychopathology and psychotherapy. Important to note is that qualitative research, as described above, refers to empirical research, not to basic or theoretical investigations. Phenomenological qualitative research in psychology has been developed using Husserlian concepts such as the *“epoché”* and the “phenomenological reduction,” and precisely on the use of such conceptualizations is where most of the current discussion has been placed. The article, therefore, will not attempt to provide a broad understanding of the phenomenological tradition. Instead, it will focus on a more specific discussion of methodological issues concerning the empirical application of phenomenology in qualitative research in psychiatry and psychology, and Husserl’s methodology in particular. To do so, we first need to agree that the application of phenomenology to empirical research in psychiatry and psychology employing interviews is qualitative, not quantitative. In a strict sense, quantitative methodology based on frequency and scales of severity of the patients’ anomalous experience, although necessary for the statistical validation of the interviews, goes beyond the scope of phenomenology. According to the phenomenological approach, mental disorders cannot be reducible to a cerebral organic basis, nor to numbers, as they are not entities *per se* but psychopathological configurations that can be identified in the diagnostic process of interaction between a clinician and a patient (Fuchs, 2010a; Pallagrosi et al., 2014; Pallagrosi and Fonzi, 2018; Gozé et al., 2019). Consequently, phenomenological interviews are designed to address not objective, but subjective data, namely the *what it is like* of patients’ anomalous experiences. In this way, the patients’ descriptions of their subjective experiences are not conceived as “static” entities, but, rather, as part of dynamically, open-ended developing processes and interpretations (Martiny, 2017).

## What

What makes an interview “phenomenological”? What are phenomenological interviews used for in empirical research in psychiatry and psychology?

Medical psychiatric diagnosis relies on standardized manuals providing a description of the apparent symptomatology and mostly excludes any assessment of subjective experience (Mishara, 1994; Parnas and Zahavi, 2002; Fuchs, 2010a). Under this approach, research in psychiatry has mainly developed from a third-person perspective, using the methods of the physical and natural sciences. Biomedical psychiatry has prioritized the use of quantitative methods and statistical analysis, whereas the value of qualitative in-depth analysis has been underestimated. The preferred experimental design has been the randomized controlled trial to demonstrate the efficacy of treatments involving psychoactive drugs (Deacon, 2013; Deacon and McKay, 2015). An alternative conceptual model to this comes from the phenomenological tradition of psychopathology. In order to understand and conceptualize the anomalous experience of a given mental illness, the phenomenological diagnosis highlights the importance of assessing patients’ subjectivity. Over the last two decades, phenomenological interviews have been developed to complement standardized diagnostic systems such as Diagnostic and Statistical Manual of Mental Disorders (DSM-5) (American Psychiatric Association, 2013) and International Statistical Classification of Diseases and Related Health Problems (ICD-10) (World Health Organization, 2012). The most important phenomenological interviews are the Examination of Anomalous Self-experience (EASE, Parnas et al., 2005) and its supplement, the Examination of Anomalous World Experience (EAWE, Sass et al., 2017). These interviews have been inspired by the Husserlian tradition and have incorporated classical descriptions of phenomenological psychopathology (particularly from Blankenburg, Conrad, and Minkowski, among other authors). Their semi-structured design allows for an in-depth examination of the patients’ subjective experiences within formal structures, such as corporeality, temporality, spatiality, and intersubjectivity. In this way, the descriptive task is not carried out on a totally random basis, as the interviews have specific domains and items that have already been established to guide the examination of the patient’s experience. EASE and EAWE were developed with the chief purpose of exploring and better understanding patients’ experiential and behavioral manifestations of schizophrenia spectrum disorders. These interviews offer comprehensive descriptions of disorders of the pre-reflexive self or *ipseity* (Sass, 1992; Parnas and Handest, 2003; Sass and Parnas, 2003; Parnas and Sass, 2008; Raballo et al., 2009; Fuchs, 2010b, 2013a; Sass et al., 2018). Indeed, EASE and EAWE have had great international impact in clinical practice and empirical research in psychiatry and psychology, and EASE has been translated into more than 10 languages, among them German, Danish, Spanish, Italian, and French.

EASE and EAWE describe aspects of the patients’ anomalous experience that are not only relevant for diagnostic but also for psychotherapeutic purposes, as they can be useful as tools in both psychotherapeutic settings and in psychotherapy research. However, phenomenological psychopathology has focused primarily on the issue of psychiatric diagnosis, while the treatment of mental illness has remained less developed. Only in recent years has the treatment of mental illness become the focus of stronger research interest, directly involving the practice of psychotherapy (Fuchs et al., 2019). For its part, although not rooted in phenomenology, body-oriented therapy has been linked to a phenomenological framework, as it provides empirical evidence for embodiment-approach conceptualizations (Fuchs 2005; Fuchs and Schlimme, 2009; Koch and Fuchs, 2011; Fuchs and Koch 2014). The embodiment approach regards schizophrenia as a fundamental disturbance of embodiment, namely a “disembodiment,” that entails a diminishment of the basic sense of self, a disruption of implicit bodily functioning and, as a result, a disconnection from intercorporeality with others. A range of empirical research into body-oriented therapy has been carried out in the field of phenomenological psychopathology. Empirical evidence of the effectiveness of body-oriented therapy for schizophrenia has been obtained from quantitative research carried out with manualized interventions (Röhricht and Papadopoulos, 2010) and using randomized controlled trials to measure outcomes (Martin et al., 2016). Recent research has incorporated phenomenological interviews to describe therapeutic change processes in body-oriented therapy for schizophrenia, thus explaining the relationship between processes and outcomes (Galbusera et al., 2018). Unsurprisingly, the phenomenological interviews revealed an understanding of change as a recovery of a “sense of self” in patients with schizophrenia (Galbusera et al., 2019).

The conceptualization of schizophrenia as a disorder of the self is shared by a number of philosophical and clinical approaches: it is not exclusive to phenomenological psychiatry (Parnas and Henriksen, 2014). So, in much the same way as body therapy has been “converted” to phenomenology, any other psychotherapeutic approach might well incorporate “front-loaded phenomenology,” in the sense of the possibility of being linked to the phenomenological framework. This is especially the case when the effectiveness of psychotherapy has been widely evidenced and recognized independently of its theoretical framework (Campbell et al., 2013). For instance, narrative/dialogical psychotherapy addressing schizophrenia as a disorder of the self might be consistent with the phenomenological conceptualization and could even serve as a complement for body-oriented therapy. In fact, EASE’s and EAWE’s rich descriptions provide evidence that patients with schizophrenia are able to communicate their experience in a comprehensive narrative form, which is quite contrary to Martin et al.’s (2016) claim that verbal dialogue can be difficult in patients with severe mental disorders. A suitable alternative might be the “metacognitive model” (Lysaker et al., 2018a). Under this model, deficits in metacognition undermine the availability of a sense of self, others, and the world, making it difficult to provide an adequate response to everyday-life situations. To deal with this, the so-called metacognitive reflection and insight therapy (MERIT) has been designed to target metacognition and recover the availability of a sense of self in the patients’ experience (Lysaker et al., 2018b). Precisely because contemporary phenomenological psychiatry places particular emphasis on the bodily and pre-reflective level of experience, the use of phenomenological interviews to explore change process in MERIT might reveal interesting relationships between pre-reflexive and reflective forms of self-experience.

Does psychotherapy needs be rooted in the phenomenological tradition in order to be called “phenomenological?” Here we are talking about enterprises such as Freud’s psychoanalysis or Binswanger’s existential analysis/daseinsanalysis. Such an enterprise requires a well-achieved and comprehensive conceptualization of phenomenological psychopathology as well as a consequent psychotherapeutic intervention rooted in the same phenomenological conceptualization. Certainly, psychotherapy does not need to be rooted in phenomenology, although this enterprise, not a minor one, might be worth undertaking. Yet, the very essence of phenomenological psychotherapy is to remain faithful to the patient’s self-experience and their constitutive vulnerability (Fuchs, 2013b; Irarrázaval, 2013, 2018; Irarrázaval and Sharim, 2014; Škodlar and Henriksen, 2019). Consequently, the development of integrative models of psychotherapy both bodily and narrative/dialogical addressing the patients’ experience of vulnerability is definitely a future challenge.

## Why

Why do we carry out phenomenological interviews with patients? Is merely contrasting phenomenological hypotheses or concepts enough to justify the patients’ involvement?

The justification for empirical research employing phenomenological interviews is extremely important, especially when persons with mental illnesses are involved. It is not only a matter of gathering data from the patients’ experience but also one of what to do with this data and, in the end, what for. It is an ethical issue concerning the impact phenomenological interviews might have on patients interviewed. Any interview aimed at exploring the experience of a patient always involves some kind of intervention, so even when applied by accredited experienced clinicians, an ethical justification is required. Arguments before ethics committees that phenomenological interviews are beneficial and do not worsen patients’ instability need to be convincing. Recalling and enacting in patients disturbing experiences we aim to grasp is certainly an intervention that needs justification. Obviously, phenomenological interviews are not psychotherapeutic interventions in themselves – that is, the dialogue in psychotherapy is not an interview – but they can be justified on the grounds similar to those usually employed by psychotherapy: the possibility of sharing anomalous experiences through an accepting and understanding communication helps patients to recover a sense of familiarity with their experience, thus reducing their sense of self-alienation. Furthermore, by means of the descriptive tasks called for in the semi-structured interviews, patients improve their articulation of anomalous experiences, which might have been otherwise overlooked, neglected, or even remain ineffable for them (Zahavi and Martiny, 2019).

Phenomenological interviews have been simply defined as falling within the framework of an interview “which is informed by insights and concepts from the phenomenological tradition and (which) in turn informs a phenomenological investigation” (Høffding and Martiny, 2016, p. 540). However, phenomenological interviews involving patients with mental illness should not only be consistent with insights and concepts from the phenomenological tradition of philosophy and psychopathology but, most importantly, they must make explicit their contribution to both diagnosis and psychotherapy. While a biomedical psychiatric diagnosis is ultimately oriented toward finding a suitable pharmacological treatment, a phenomenological diagnosis is ultimately oriented toward providing a treatment based on the experiential dimension of a given mental illness. The interest of a psychotherapist goes beyond the psychiatric diagnostic emphasis by approaching the patient as a whole person, aiming to understand the anomalies of experience within his/her social, cultural, and historical context. This broader, psychological, approach enables an understanding not only of how patients make sense of their anomalous experiences but also of how symptoms manifest themselves within the patients’ immediate life context, as well as how a certain mental illness configures itself along the patients’ history of meaningful interactions with others (Irarrázaval and Sharim, 2014; Irarrázaval, 2018). However, in spite of the importance given to the analysis of the patients’ biography by several authors from the phenomenological tradition of psychopathology (Jaspers, Binswanger, and Blankenburg, among other authors), “biographical methods,” originally developed for sociological research in the influential “Chicago School” (Bornat, 2008), have not been sufficiently incorporated in current phenomenological empirical research in psychiatry and psychology.

## How

How should we conduct phenomenological interviews with patients? How can we properly perform analysis in empirical phenomenological research in psychiatry and psychology?

A phenomenological interview involves a second-person situation, in which the dialogical communication with the patient is crucial. No matter how strange or unrealistic the patients’ anomalous experiences might appear to the interviewer, an attitude of professional competence and familiarity is necessary (Nordgaard et al., 2013). For the patient, anomalous experiences are actually lived experiences despite their lack of commonsensical validity. Hallucinations and delusions are, like nonpsychotic experiences, first-personally given, which means that they have a solipsistic validity. This is one of the reasons why it is difficult, especially in psychotic phases, for patients to come to terms with the fact that what they actually experience is not credible or real in the eyes of others, and even abnormal or pathological in the eyes of the clinician. Clearly, the interviewer’s role is not to confront or contradict this lack of commonsensical validity, but simply to grasp the experiences as they appear to the patients. In other words, the interviewer conducts the interview with an attitude of empathetic understanding. Empathy should not be reduced to an attempt to understand the patient in a “representational” manner, in the sense that it does not refer to the interviewer’s own experience of processing (imitating, thinking, or imagining) the patient’s subjectivity (Irarrázaval, 2019). Empathy is the condition of possibility for the “subject-subject” relationship (Zahavi, 2015). That is to say, empathy is a distinct mode of other-directed intentionality that permits the unfolding of the patient’s experience, approached as a unique other person. In this sense, empathic understanding permits the unfolding of the *what it is like* of the patient’s anomalous experience.

In phenomenological interviews, why-like questions lead patients to respond with causal explanations of the anomalies of their experience or diagnosed mental illness, such as judgments, beliefs, theoretical constructions, etc., For their part, how-like questions guide patients to describe the way in which they live their experience, that is, the way in which the anomalies actually appear to the patients in their experience. To put it another way, both types of questions lead patients to talk about experiential contents, but in different ways: causal attributions in the former, and appearances in the latter. Causal attributions are by no means irrelevant aspects of the patient’s experience not worth addressing in the interview. The way in which patients’ attribute causes to their anomalous experience or mental illness can also provide valuable information for both diagnosis and psychotherapy. Moreover, the relationship between causal attributions and appearances is certainly valuable, as it entails a circular, dynamic process in which both orders of experiencing constantly influence one another. However, the gathering of phenomenological data is generally not aimed at obtaining causal explanations or attributional reports, as in the case of cognitive psychology, but mainly at exploring aspects of experience that how-like questions are designed to unfold.

Turning to data analysis, it has been said that phenomenology is interested in describing the formal structure of the experience rather than its content (Gallagher and Zahavi, 2008), but what does this actually mean? It seems difficult to imagine an experience as a mere structure without any content. Moreover, it is not possible to establish a category of experience that has not been previously built upon any content analysis. In qualitative studies, categories are built upon the basis of prior content analysis; both hypotheses and categories are developed as the study progresses and emerge from the data itself (Morrow, 2005; Maxwell, 2012), so-called “iterative process” (Barbour and Barbour, 2003). EASE and EAWE were built collecting first-person descriptions by a significant number of patients (around 100 each), which allowed for their statistical validation. However, only a fairly general description has been provided of how EASE’s domains and items were developed: singular contents of anomalous experience are conceptualized and interconnected within a comprehensive system of meaningful structural wholes or *Gestalts*, leading to the “core” underlying psychopathological configuration (Nordgaard et al., 2013). A recent qualitative study on the responses to the two scales highlights the specificities of the phenomena described by EASE and EAWE, indicating that disturbances of world experience are fundamentally less unitary, while the experience of the self presents a more coherent and unitary *Gestalt* (Englebert et al., 2019).

Beyond the statistical validation of the interviews, replication is needed in other clinical samples and cultures to support previous findings and provide added evidence when compared with multiple clinical groups and cross culturally. However, if the focus of the analysis is placed merely on formal structural aspects, then when applying EASE and EAWE to new patients, we will not find domains or categories different from those already defined. To put it differently, quantitative replication of EASE or EAWE in other samples would barely lead to any new knowledge, because already established domains and items tend to constrain the patients’ responses. So, particularly in terms of their potential contribution to psychotherapy, the best contribution that could be made from applying EASE and EAWE to new patients would result from a content analysis of the patients’ reports. However, one key question concerning these interviews’ replication remains unanswered: Which is the most appropriate qualitative method for analyzing the patients’ descriptions?

The empirical application of Husserl’s phenomenological method outside the strict scope of philosophy still is a topic of ongoing debate in both philosophy and the cognitive sciences. According to Zahavi (2019a,b,c), in philosophy, the main goal of phenomenology is not purely descriptive or attentive to how things appear to the subject; it focuses neither on the subject nor on the object, but on the correlation between them. In this context, the term *epoché* is used to refer to suspending or putting between parentheses a “naïve” or “natural” attitude toward reality in order to reflect upon fundamental ontological questions, thus adopting a critical stance on the conception of reality as mind-independently given. *Epoché*, usually described as putting “in brackets” the prejudices and theoretical assumptions of the interviewer (Fischer, 2009), in order to access phenomena as they appear in the subject’s experience, has little to do with the original philosophical method. This does not imply that bracketing our prejudices and theoretical assumptions would not be desirable to avoid bias when conducting phenomenological interviews or analyzing data (we can find several techniques for doing so). It is also not so important to calling such bracketing *epoché*, as long as we have a basic notion of Husserl’s original sense of the term.

Phenomenology has been applied in empirical research not only in psychiatry and psychology, but also in other healthcare-related disciplines, such as nursing studies (Zahavi and Martiny, 2019). Nevertheless, the different forms in which phenomenology has been applied in these disciplines have been also controversial due to their divergence from the original Husserlian philosophical method (Zahavi, 2019b,d). For instance, some have questioned whether the method of analysis proposed by Giorgi (2009, 2012), “descriptive phenomenological psychological method,” should be considered “phenomenological” or given another label. This method is aimed at the establishment of inclusive categories resulting from the content analysis of subjects’ descriptions. In fact, Giorgi’s method of content analysis seems closer to an adapted form of “eidetic variation” and quite different to the original Husserlian sense of the *epoché*, because it basically consists of summarizing the content of the interview transcript by deleting its redundancies, in order to reveal invariables or essences in “meaning” (see Irarrázaval, 2015). Eidetic variation is a conceptual analysis that, by imagining a phenomenon as being different from how it currently is, leads to the isolation of its essential features or aspects, in the sense that such features or aspects cannot be varied or deleted without preventing the phenomenon from being the kind of phenomenon that it is (Parnas and Zahavi, 2002). Another example of a so-called applied phenomenological method is “microphenomenology” (Petitmengin et al., 2018; Depraz, 2020). This method, like Giorgi’s, also diverges from the original Husserlian philosophical method. In addition to the method of analysis, micro-phenomenology includes some “principles” regarding the interview. Microphenomenological analysis seeks to identify generic pre-reflexive structures from descriptions of “singular” lived experiences. The pre-reflexive aspect of experience is conceived as experientially “unnoticed,” in the sense that it is not immediately accessible to reflective consciousness and verbal description. However, at least in the way Petitmengin et al. (2018) present it, what results from the analysis seems to be more a description of the figurative aspects or features of the object rather than experiential structures of the subject (for example, size, shape, temperature, color, etc.,).

## Discussion

Whether to find evidence supporting already-existing insights and concepts or to make it possible for new insights and concepts to emerge from the data itself, phenomenological empirical research must take on board patients’ accounts of their subjective experience. Phenomenological interviews should present clear guidelines on both how to conduct them and the qualitative methods employed in analyzing patients’ subjective experiences. The research report should follow standards for presenting qualitative research (O’Brien et al., 2014). Still, the most challenging aspect of phenomenological empirical research in psychiatry and psychology is the proper method for analyzing patients’ reports. Neither the original Husserlian question of phenomenological philosophizing nor the phenomenological method of philosophical analysis appears appropriate for empirical application. There seems to be a gap between the phenomenological philosophical method and its empirical versions.

Phenomenological philosophy, psychiatry, and psychology have different aims and practical implications. This implies that the methods used in each of these research fields are necessarily different, since they serve as a means to achieve the different aims pursued by each of the corresponding disciplines. In philosophy, the phenomenological method serves as a means to reflect upon fundamental ontological questions regarding our active subjective involvement in the constitution of the world. However, in phenomenological psychiatry and psychology, the methods serve as a means to achieve more precise, complete, and differential diagnoses, with the aim of improving psychotherapy and, ultimately, patients’ well-being. Nevertheless, regardless of their divergence from the original philosophical method, Georgi’s method of content analysis (to a greater extent), and “microphenomenology” (to a lesser extent), have been quite influential, precisely because of their attempt to bridge this gap, providing a response to the need for a phenomenological method for qualitative research.

An entirely different way of dealing with this problem would not be to seek empirical adaptations of the original phenomenological method inherent in philosophy, nor to limit phenomenology to a mere descriptive task of subjective experience, but to make phenomenology a theoretical framework for empirical research, and even more, a transcendental paradigm. Although its method is certainly fundamental to it, phenomenology should not be reduced to its methodology. Phenomenology is a comprehensive theoretical framework that has been developed on the basis of serious conceptual and empirical research into the subject-world correlation (Zahavi, 2019a), including studies of formal structures of experience (spatiality, temporality, corporeality, intersubjectivity, and historicity), research into the modes of intentionality (perception, agency, phantasy, memory, emotions, and empathy), and psychological analyses of meaning-making processes in social interactions. Additionally, despite the different aims and methods involved, just as in phenomenological philosophy, in phenomenological psychiatry and psychology the core philosophical commitment regarding a critical stance on the conception of reality as mind-independently given is fundamental (Zahavi, 2017, 2019e). Does psychiatry and psychology really need the Husserlian method to adopt the phenomenological attitude toward the conception of reality as mind-dependent? No, because this core philosophical commitment already constitutes the basis of a transcendental paradigm in phenomenological psychiatry and psychology.

Mainstream psychiatry has been developed within a natural-scientific paradigm. From the positivist viewpoint of psychiatry, the notion of normality is defined with regard to the degree of correspondence between subjective experience and objective reality. Consequently, abnormality is defined in terms of its degree of deviation from an objective reality that provides the evidence for commonsensical validity. For its part, phenomenological psychopathology approaches mental phenomena in terms of a phenomenological analysis of the patient’s subjectivity, placing the focus on the conditions of possibility of human experience in general, beyond it being diagnosed as abnormal according to common standards of objectivity. For instance, in current diagnostic systems, psychosis is diagnosed by the presence of hallucinations and delusions, as defined by a “natural attitude” that takes for granted the validity of an objective given reality. In DSM-5, hallucination is defined as a perception without object (or an error of perception) and delusion as a false belief of reality (American Psychiatric Association, 2013). In contrast, from a phenomenological approach, a disturbance is not approached in terms of the clinician’s evidence of the inexistence of the object of perception or the lack of external evidence of the patient’s belief, but rather in terms of an analysis of the particular mode of intentionality that constitutes the hallucination or delusion as such. In other words, the clinician is concerned with a phenomenological analysis of the patient’s subjectivity, addressing with empathic understanding the patient’s “self-evidence” or “solipsistic truth,” correlated with the experience of hallucination or delusion, respectively. Indeed, the “external” inexistent object should provide for the clinician with evidence that hallucination is not perception, as it is impossible to have a perception without a directly present object.

Consequently, it would be misleading to conceive of hallucination as something to do with perception at all. Instead, hallucinations would have more to do with the phenomenology of fantasy, whose distinctive character is to “re-present” an object of perception that is not directly present, but absent from the actual field of perception. According to Cavallaro (2017), it is not the presentation/re-presentation dichotomy, but what Husserl calls “ego-splitting” (*Ichspaltung*) that is crucial to distinguishing when experiencing the “quasi perception” produced by fantasy and not a perception as such. Ego-splitting makes possible the experience of the “as if” fictive character of self-awareness when fantasizing. However, when hallucinating, the patient experiences his/her own thoughts, anticipations, or imaginations just as in original experiences of perception. So, it may be posited that it is precisely this lack of the “as if” self-awareness of the “quasi perception” that lies at the core of psychosis. Such a theory would require further phenomenological research to draw more distinctions between the nature of hallucination in contrast to that of fantasy, as well as regarding other modalities of experiencing which do not have an intentional object directly present, such as anticipations, thoughts, memories, and dreams. Still, introducing the concept of “ego-splitting” as non-pathological might be challenging to traditional psychiatric concepts, especially with regard to schizophrenia.

Finally, the phenomenological attitude should not be conceived of as being like any other attitude; it is obviously not literally an attitude. The phenomenological attitude is a paradigmatic commitment of a non-pregiven reality. This core philosophical commitment is particularly important because it entails a quite unique approach to mental illness, including different conceptualizations of psychopathology, diagnosis, normality, empathy, and psychotherapy, thus leading qualitative empirical research in psychiatry and psychology toward new horizons. Moreover, the notion of suspending the natural attitude to approaching reality (including all kinds of phenomena) lies at the heart of the phenomenological framework for anyone claiming to be a phenomenologist, whether conceptual or empirical, and regardless of other particular methods and topics of study. In this way, the phenomenological attitude might be conceived of the basis of a transcendental scientific paradigm for qualitative research in psychiatry and psychology. This latter claim, which supports the idea that phenomenological psychology – in order to be properly phenomenological – must become transcendental, and the phenomenological conceptualization of hallucination as pathology of fantasy provide challenging directions for future research.

## Author Contributions

The author confirms being the sole contributor of this work and has approved it for publication.

### Conflict of Interest

The author declares that the research was conducted in the absence of any commercial or financial relationships that could be construed as a potential conflict of interest.
