# Erratum: A Phenomenological Paradigm for Empirical Research in Psychiatry and Psychology: Open Questions

**DOI:** 10.3389/fpsyg.2020.01989

**Published:** 2020-07-23

**Authors:** Frontiers Production Office

**Affiliations:** Frontiers Production Office, Frontiers Media SA, Lausanne, Switzerland

**Keywords:** applied phenomenology, methodology, qualitative research, psychiatry and psychology, phenomenological interviews

Due to an error in the typesetting process, in the last paragraph of the Introduction, the term “*epoché*” was erroneously replaced by the term “*what it is like*.” A correction has therefore been made to the **Introduction** section, paragraph four:

“This article will address the “What,” “Why,” and “How” of phenomenological interviews, reviewing recent empirical research in the field of phenomenological psychopathology and psychotherapy. Important to note is that qualitative research, as described above, refers to empirical research, not to basic or theoretical investigations. Phenomenological qualitative research in psychology has been developed using Husserlian concepts such as the “*epoché”* and the “phenomenological reduction,” and precisely on the use of such conceptualizations is where most of the current discussion has been placed. The article, therefore, will not attempt to provide a broad understanding of the phenomenological tradition. Instead, it will focus on a more specific discussion of methodological issues concerning the empirical application of phenomenology in qualitative research in psychiatry and psychology, and Husserl's methodology in particular. To do so, we first need to agree that the application of phenomenology to empirical research in psychiatry and psychology employing interviews is qualitative, not quantitative. In a strict sense, quantitative methodology based on frequency and scales of severity of the patients' anomalous experience, although necessary for the statistical validation of the interviews, goes beyond the scope of phenomenology. According to the phenomenological approach, mental disorders cannot be reducible to a cerebral organic basis, nor to numbers, as they are not entities *per se* but psychopathological configurations that can be identified in the diagnostic process of interaction between a clinician and a patient (Fuchs, 2010; Pallagrosi et al., 2014; Pallagrosi and Fonzi, 2018; Gozé et al., 2019). Consequently, phenomenological interviews are designed to address not objective, but subjective data, namely the *what it is like* of patients' anomalous experiences. In this way, the patients' descriptions of their subjective experiences are not conceived as “static” entities, but, rather, as part of dynamically, open-ended developing processes and interpretations (Martiny, 2017).

The publisher apologizes for this mistake. The original article has been updated.
